# Heat and mass transfer of micropolar liquid flow due to porous stretching/shrinking surface with ternary nanoparticles

**DOI:** 10.1038/s41598-023-29469-0

**Published:** 2023-02-21

**Authors:** G. P. Vanitha, U. S. Mahabaleshwar, M. Hatami, Xiaohu Yang

**Affiliations:** 1 Department of Mathematics, Siddaganga Institute of Technology, Tumkur, 572103 India; 2 Department of Studies in Mathematics, Davangere University, Shivagangothri, Davangere, 577007 India; 3 Department of Mechanical Engineering, Esfarayen University of Technology, Esfarayen, Iran; 4 CNOOC (Tianjin) Pipeline Engineering Technology Co., Ltd., Tianjin, China

**Keywords:** Mechanical engineering, Applied mathematics, Computational science, Nanomedicine, Other nanotechnology

## Abstract

The present investigation is carried out to predict the flow characteristics of a micropolar liquid that is infused with ternary nanoparticles across a stretching/shrinking surface under the impact of chemical reactions and radiation. Here, three dissimilarly shaped nanoparticles (copper oxide, graphene and copper nanotubes) are suspended in H_2_O to analyse the characteristics of flow, heat and mass transfer. The flow is analysed using the inverse Darcy model, while the thermal analysis is based on the thermal radiation. Furthermore, the mass transfer is examined in light of the impact of first order chemically reactive species. The considered flow problem is modelled resulting with the governing equations. These governing equations are highly non linear partial differential equations. Adopting suitable similarity transformations partial differential equations are reduced to ordinary differential equations. The thermal and mass transfer analysis comprises two cases: PST/PSC and PHF/PMF. The analytical solution for energy and mass characteristics is extracted in terms of an incomplete gamma function. The characteristics of a micropolar liquid are analysed for various parameters and presented through graphs. The impact of skin friction is also considered in this analysis. The stretching and rate of mass transfer have a large influence on the microstructure of a product manufactured in the industries. The analytical results produced in the current study seem to be helpful in the polymer industry for manufacturing stretched plastic sheets.

## Introduction

The theoretical study of micropolar fluids is a viscous fluid that suspends inflexible tiny particles that are highly irregular, rotate and spin slightly about their own axes. Fluids like blood, paint, lubricant fluids, anisotropic fluids, polymers, animal blood, complex biological structures are a few examples of microfluid that have significant applications in industries. Eringen^1^ is the pioneer who proposed the microfluidic theory. In this theory, a new constitutive equation and a new micro-rotation material independent of the vector field are added to the Navier–Stokes equation. Eringen^2^ expanded on his earlier research by providing a generalised theory of thermal micropolar fluid. Guram and Smith^3^ studied micropolar fluid stagnation flows with strong and weak synergy. Sankara et al.^4^ investigated the micropolar fluid flow across a stretching sheet using the highly convergent Homotopy method to obtain the numerical results. Several earlier researches, including those by Hady^5^, Heruska^6^ and Chiam^7^, are motivated by the potential significance of micropolar boundary layer flow in industrial applications. Since then, numerous authors^8–15^ have investigated the impacts of different physical parameters on micropolar fluid, including magnetohydrodynamics (MHD), Joule heating, radiation, chemical reaction and viscous dissipation.

On the other hand, numerous studies have examined the impact of nanoparticle inclusion on the properties of heat transport in various physical situations. A nanofluid is a fluid composed of highly thermally conductive nanoparticles suspended in a base fluid. Due to the metallic nanoparticles suspended in the fluid, the nanofluid has a greater thermal conductivity than a typical fluid, is chemically stable, and exhibits improved heat transfer rates, nanofluid has uses in the petroleum industries, pharmaceutical industry, and many other fields. Dulal Pal^16,17^ analysed the hall effects and stagnation point flow of nanofluid over a stretching/shrinking sheet. Krishnandan et al.^18^ examined computationally MHD nanoparticles flow over a shrinking sheet under the impact of chemical reactions and applied heat approaching the stagnation point of micropolar fluid, their findings reveals that when the Biot number increases, the temperature of the nanofluid and the distribution of nanoparticles both increases. Alizadeh et al*.*^19^ investigated the heat transfer among permeable materials and micropolar nanofluid flow walls exposed to a magnetic field and heat radiation. Bilal^20^ study involves mixed convective micropolar nanoparticles flowing over an upward sheet with slip and ohmic dissipation. The investigation on MHD micropolar nanofluid flow enclosed by two surfaces with radiation and hall current was carried out by Saeed et al.^21^. Rafique et al.^22^ discussed micropolar nanofluid hydromagnetic flow. Patnaik et al.^23^ used ADM-Pade computation technique to analyse the mixed convection flow of MHD micropolar nanofluid flow with chemical reaction past a porous stretching surface. Aslani et al.^24^ conducted a study on MHD micropolar fluid flow across a penetrable stretching/shrinking sheet with a radiation effect. Gadisa et al.^25^ used a numerical technique to analyse the effect of couple stress of micropolar nanofluid flow by formulating the problem using a non-Fourier’s-law heat flux model.

Many researchers like Shaheen et al.^26^, Rojaa et al.^27^, Mahabaleshwar et al.^28,29^ investigated the micropolar nanofluid flow considering MHD, mass transpiration, viscous dissipation, thermal radiation, heat source/sink, chemical reaction. A two-dimensional motion past a porous linear stretching/shrinking sheet and mass transfer of the non-Newtonian flow with Cu-Al_2_O_3_ hybrid nanoparticles suspension is examined by Mahabaleshwar et al.^30^. An analysis of entropy generation using Darcy-Forchheimer model with hybrid nanofluid is reported by Gopinath and Dulal Pal^31^. The numerical analysis of ternary hybrid nanoparticles motion in between the parallel plates is illustrated by Bilal et al.^32^. Bhattacharyya et al.^33^, Heruska et al.^34^, Mohammadein et al.^35^, Dulal^36,37^ and Mahmoud^38^ explained the thermal radiation effects on micropolar fluid past a shrinking/stretching sheet.

In this study, we analyse the impact of heat and mass transfer on a micropolar fluid suspended with ternary nanoparticles as it passes past a porous stretching/shrinking sheet. Dual solutions for the momentum and microrotation are obtained using analytical technique. Heat and mass transfer are analysed for two different boundary conditions, and solutions are evaluated in terms of an incomplete gamma function. The characteristics of the flow field and skin friction are discussed and presented through graphs. The current paper explanation starts with a theoretical analysis in “Theoretical analysis” section. “Methodology and non-dimensional variables” section contains methodology, an analysis of the flow, heat and mass fields is mentioned in “Solution analysis” section. Further, “Result analysis” section mentions the result analysis followed by concluding remarks in “Concluding remarks” section.

## Theoretical analysis

**Figure 1:**
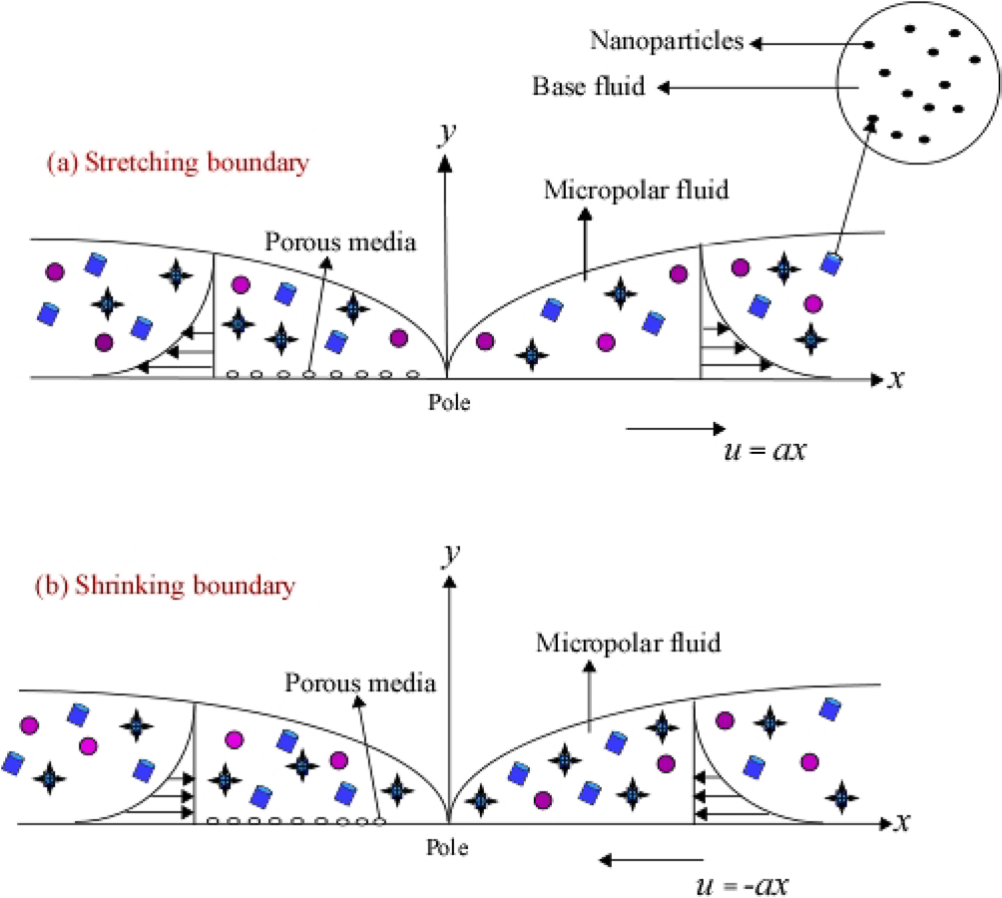
Schematic representation of the stretching/shrinking boundary.

The steady, laminar, two-dimensional boundary layer flow of micropolar fluid infused with ternary nanoparticles of different shapes was under study to analyse the behaviour of the flow, energy, and mass transfer caused by a stretching/shrinking sheet under the influence of thermal radiation and chemical reactions as explained in Fig. 1.

The considered fluid has high porosity in the porous medium ($\varepsilon = 1$). The fundamental equations of the flow field (Nagaraju et al.^39^) are modelled as follows:

### Continuity equation

1 $$\frac{\partial u}{{\partial x}} + \frac{{\partial {\text{v}}}}{\partial y} = 0,$$

### Momentum equation

2 $$u\frac{\partial u}{{\partial x}} + {\text{v}}\frac{\partial u}{{\partial y}} = - \frac{1}{{\rho_{tnf} }}\frac{\partial p}{{\partial x}} + \left( {\nu_{tnf} + \frac{{\kappa_{tnf} }}{{\rho_{tnf} }}} \right)\left( {\frac{{\partial^{2} u}}{{\partial x^{2} }} + \frac{{\partial^{2} u}}{{\partial y^{2} }}} \right) - \frac{{\mu_{tnf} }}{{\rho_{tnf} k_{tnf} }}u + \frac{{\kappa_{tnf} }}{{\rho_{tnf} }}\frac{\partial \omega }{{\partial y}},$$

3 $$u\frac{{\partial {\text{v}}}}{\partial x} + {\text{v}}\frac{{\partial {\text{v}}}}{\partial y} = - \frac{1}{{\rho_{tnf} }}\frac{\partial p}{{\partial y}} + \left( {\nu_{tnf} + \frac{{\kappa_{tnf} }}{{\rho_{tnf} }}} \right)\left( {\frac{{\partial^{2} {\text{v}}}}{{\partial x^{2} }} + \frac{{\partial^{2} {\text{v}}}}{{\partial y^{2} }}} \right) - \frac{{\mu_{tnf} }}{{\rho_{tnf} k_{tnf} }}{\text{v}} + \frac{{\kappa_{tnf} }}{{\rho_{tnf} }}\frac{\partial \omega }{{\partial x}}\,,$$

### Microrotation equation

4 $$u\frac{\partial \omega }{{\partial x}} + v\frac{\partial \omega }{{\partial y}} = \frac{{\gamma_{tnf} }}{{J\,\rho_{tnf} }}\left( {\frac{{\partial^{2} \omega }}{{\partial x^{2} }} + \frac{{\partial^{2} \omega }}{{\partial y^{2} }}} \right) - \frac{{\kappa_{tnf} }}{{J\,\rho_{tnf} }}\left( {2\omega + \frac{\partial u}{{\partial y}}} \right) + \kappa_{tnf} \left( {\frac{\partial v}{{\partial x}} - \frac{\partial u}{{\partial y}}} \right),$$

### Energy equation

5 $$u\frac{\partial t}{{\partial x}} + {\text{v}}\frac{\partial t}{{\partial y}} = \alpha_{tnf} \left( {\frac{{\partial^{2} t}}{{\partial x^{2} }} + \frac{{\partial^{2} t}}{{\partial y^{2} }}} \right) - \frac{1}{{\left( {\rho C_{p} } \right)_{tnf} }}\frac{{\partial q_{r} }}{\partial y},$$

### Concentration equation

6 $$u\frac{\partial c}{{\partial x}} + {\text{v}}\frac{\partial c}{{\partial y}} = D\left( {\frac{{\partial^{2} c}}{{\partial x^{2} }} + \frac{{\partial^{2} c}}{{\partial y^{2} }}} \right) - K\left( {c - c_{\infty } } \right).$$

Here, $u$ and ${\text{v}}$ are the velocity components along x and y axis, respectively. $\frac{dp}{{dx}}$ is the pressure gradient. It is assumed to be zero because fluid flow is due to the stretching/shrinking of the sheet. $\omega$ is the microrotation component obtained from the vector $\vec{\omega } = (0,0,\omega )$.The terms: $\rho_{tnf}$ represents density, $\mu_{tnf}$ denotes viscosity, $\alpha_{tnf}$ stands for thermal diffusivity, $\nu_{tnf}$ is kinematic viscosity of the ternary nanoparticles micropolar liquid. $t$ and $c$ denotes thermal and solutal quantity of the liquid.$K$ denote the first order chemical reaction.

Using the aforementioned boundary layer assumptions, Eqs. (1)–(6) are reduced to the following PDEs (Sankara and Watson^40^):

7 $$\frac{\partial u}{{\partial x}} + \frac{\partial v}{{\partial y}} = 0,$$

8 $$u\frac{\partial u}{{\partial x}} + {\text{v}}\frac{\partial u}{{\partial y}} = \left( {\nu_{tnf} + \frac{{\kappa_{tnf} }}{{\rho_{tnf} }}} \right)\frac{{\partial^{2} u}}{{\partial y^{2} }} - \frac{{\mu_{tnf} }}{{\rho_{tnf} k_{tnf} }}u + \frac{{\kappa_{tnf} }}{{\rho_{tnf} }}\frac{\partial \omega }{{\partial y}},$$

9 $$u\frac{\partial \omega }{{\partial x}} + v\frac{\partial \omega }{{\partial y}} = \frac{{\gamma_{tnf} }}{{J\,\rho_{tnf} }}\left( {\frac{{\partial^{2} \omega }}{{\partial y^{2} }}} \right) - \frac{{\kappa_{tnf} }}{{J\,\rho_{tnf} }}\left( {2\omega + \frac{\partial u}{{\partial y}}} \right),$$

10 $$u\frac{\partial t}{{\partial x}} + {\text{v}}\frac{\partial t}{{\partial y}} = \alpha_{tnf} \left( {\frac{{\partial^{2} t}}{{\partial y^{2} }}} \right) - \frac{1}{{\left( {\rho C_{p} } \right)_{tnf} }}\frac{{\partial q_{r} }}{\partial y},$$

11 $$u\frac{\partial c}{{\partial x}} + {\text{v}}\frac{\partial c}{{\partial y}} = \frac{D}{{\nu_{tnf} }}\left( {\frac{{\partial^{2} c}}{{\partial y^{2} }}} \right) - K_{1} \left( {c - c_{\infty } } \right).$$

It is evident that v represents the flow field entire spin, which includes the spin of the fluid media and microstructure. Additionally, it is possible that under some circumstances, the effects of the microstructure vanish and the flow takes on the characteristics of a typical viscous flow. As a result, if we insist that ${\text{v}}$ is the angular velocity is a feasible solution, then we consider the following condition.

12 $$\gamma_{tnf} = \mu_{tnf} \left( {1 + \frac{{\kappa_{tnf} }}{{2\mu_{tnf} }}} \right)\frac{{\nu_{tnf} }}{a}.$$

where, $\gamma_{tnf}$ signifies angular rotational viscosity. The relation in Eq. (6) is explained by^41–43^.

The prescribed boundary conditions are as follows:

13 $$\left. {\begin{array}{lll} {u = U_{w} = \pm ax,} & \quad {\text{v} = - \text{v}_{w},} & \quad {\omega = - \xi \frac{\partial u}{{\partial y}},} \\ t = t_{w} \;({\text{for}}\;{\text{the}}\;{\text{PST}}), & \quad - k\sqrt {\frac{\nu }{a}} \,\frac{\partial t}{{\partial y}} = q_{w} , & \quad ({\text{for}}\;{\text{the}}\;{\text{PHF}}) \\ {c = c_{w} \,\,\,({\text{for}}\;{\text{the}}\;{\text{PSC}})\,} & \quad { - k\sqrt {\frac{\nu }{a}} \,\frac{\partial c}{{\partial y}} = m_{w} ,} & \quad {({\text{for}}\;\,{\text{the}}\;{\text{PHF}})} \\ \end{array} } \right\}{\text{at}}\;{y} = 0$$

14 $$\left. {u \to 0\,,\,\;\;\omega \to 0,\,\;\;t \to t_{\infty } \,,\;\;c \to c_{\infty } } \right\}\,\,\,\,\,{\text{as}}\,\,\,\,y \to \infty .$$

After obtaining the governing PDEs, we now proceed to the next section, which is methodology used to extract the solution via similarity transformations.

## Methodology and non-dimensional variables

The analysis of this problem is continued by employing the following non-dimensional variables:

15 $$X = \left( {\frac{a}{\nu }} \right)^{1/2} x\,,\,\,\,\,\,\,Y = \left( {\frac{a}{\nu }} \right)^{1/2} y\,,\,\,\,\,\,\,\left( {U,V} \right) = \frac{{(u,{\text{v}})}}{{\left( {a\nu } \right)^{1/2} }}\,,\,\,\,\,\,\,\,N = \frac{\omega }{a}\,,\,\,\,\,\,T = \frac{{t - t_{\infty } }}{{t_{w} - t_{\infty } }}\,,\,\,\,\,\,C = \frac{{c - c_{\infty } }}{{c_{w} - c_{\infty } }}.$$

Here we study the heat and concentration equation for two different conditions:

Temperature equation: PSH and PHF

($t_{w} - t_{\infty }$ is fixed for PSH case; $t_{\infty } = 0$; rate of change of wall heat w.r.t ‘$x$’ is neglected for PHF case)

Concentration equation: PSC and PCF

($c_{w} - c_{\infty }$ is fixed for PSC case; $c_{\infty } = 0$; rate of change of wall concentration w.r.t ‘$x$’ is neglected for PCF case)

Using Eq. (15), the governing non-linear PDEs are simplified as non-dimensional equations as follows:

16 $$\frac{\partial U}{{\partial X}} + \frac{\partial V}{{\partial Y}} = 0,$$

17 $$U\frac{\partial U}{{\partial X}} + V\frac{\partial U}{{\partial Y}} = \left( {1 + \frac{{\kappa_{tnf} }}{{\mu_{tnf} }}} \right)\left( {\frac{{\partial^{2} U}}{{\partial Y^{2} }}} \right) - \frac{{\varepsilon \,\mu_{tnf} }}{{\rho_{tnf} k_{tnf} a}}U + \frac{{\kappa_{tnf} }}{{\mu_{tnf} }}\frac{\partial N}{{\partial Y}},$$

18 $$U\frac{\partial N}{{\partial X}} + V\frac{\partial N}{{\partial Y}} = \frac{{\gamma_{tnf} }}{{J\,\rho_{tnf} \,\nu_{tnf} }}\left( {\frac{{\partial^{2} N}}{{\partial Y^{2} }}} \right) - \frac{{\kappa_{tnf} }}{{J\,\rho_{tnf} \,a}}\left( {2N + \frac{\partial U}{{\partial Y}}} \right),$$

19 $$U\frac{\partial T}{{\partial X}} + V\frac{\partial T}{{\partial y}} = \left( {\alpha_{tnf} \left( {\frac{{\partial^{2} T}}{{\partial y^{2} }}} \right) - \frac{1}{{\left( {\rho C_{p} } \right)_{tnf} }}\frac{{\partial q_{r} }}{\partial y}} \right)\frac{1}{{\nu_{tnf} }},$$

20 $$U\frac{\partial C}{{\partial X}} + V\frac{\partial C}{{\partial Y}} = \frac{D}{{\nu_{tnf} }}\left( {\frac{{\partial^{2} C}}{{\partial Y^{2} }}} \right) - K_{1} C.$$

The associated boundary conditions are:

21 $$\left. {\begin{array}{lll} U = \pm X, & \quad V = V_{w}, & \quad N = - \xi \frac{\partial U}{{\partial Y}} \\ T = 1\,\,(\text{PST}) & \quad \frac{\partial T}{{\partial Y}} = - 1\,\,(\text{PHF}) & {} \\ C = 1\,\,(\text{PSC}) & \quad \frac{\partial C}{{\partial Y}} = - 1\,\,(\text{PMF}) & {} \\ \end{array} } \right\}{\text{at}}\;{Y} = 0$$

22 $$U = 0\,,\,\;N = 0\,,\,\;\,T \to T_{\infty } \,,\,\;\;C \to C_{\infty } \,\,\,\,\,{\text{as}}\,\,\,\,\,\,Y \to \infty .$$

In this paper, the nanoparticles with spherical and non-spherical shapes are used (cylindrical and platelet). When particles are dispersed in liquid, Suganthi et al.^44^ found that particle form has an impact on how the particles move. Additionally, their research showed that non-spherical nanoparticles perform less well in fluid flow, translational motions, and rotational motions than spherical nanoparticles. The dimensional parameters such as thermal conductivity $\kappa_{tnf}$ , density $\rho_{tnf}$, viscosity $\mu_{tnf}$ and heat capacity $\left( {\rho C_{p} } \right)_{tnf}$ of different shaped ternary nanoparticles are considered as follows based on Table 1 data^45–47^

$$\left. \begin{aligned} & \rho_{tnf} = \left( {1 - \phi_{1} - \phi_{2} - \phi_{3} } \right)\rho_{bf} + \phi_{1} \rho_{sp1} + \phi_{2} \rho_{sp2} + \phi_{3} \rho_{sp3} , \hfill \\ & \left( {\rho C_{p} } \right)_{tnf} = (1 - \phi_{1} - \phi_{2} - \phi_{3} )\left( {\rho C_{p} } \right)_{bf} + \phi_{1} \left( {\rho C_{p} } \right)_{sp1} + \phi_{2} \left( {\rho C_{p} } \right)_{sp2} + \phi_{3} \left( {\rho C_{p} } \right)_{sp3} , \hfill \\ & \left( {\rho C_{p} } \right)_{tnf} = (1 - \phi_{1} - \phi_{2} - \phi_{3} )\left( {\rho C_{p} } \right)_{bf} + \phi_{1} \left( {\rho C_{p} } \right)_{sp1} + \phi_{2} \left( {\rho C_{p} } \right)_{sp2} + \phi_{3} \left( {\rho C_{p} } \right)_{sp3} , \hfill \\ & \mu_{tnf} = \frac{{\mu_{nf1} \phi_{1} + \mu_{nf2} \phi_{2} + \mu_{nf3} \phi_{3} }}{\phi }, \hfill \\ & \kappa_{tnf} = \frac{{\kappa_{nf1} \phi_{1} + \kappa_{nf2} \phi_{2} + \kappa_{nf3} \phi_{3} }}{\phi } \hfill \\ & {\text{and}} \hfill \\ & \phi = \phi_{1} + \phi_{2} + \phi_{3} . \hfill \\ \end{aligned} \right\},$$

**Table 1:** Thermo-physical properties^48–51^.

Sl. no.	Source	Base fluid and nano-particles	$\rho$	$\kappa$	$C_{p}$	Shape
1	^48^	Water H_2_O	997.1	0.613	4180	-
2	^49^	Graphene	2200	5000	790	Platelet
3	^50^	Copper oxide (CuO)	6320	40	765	Spherical
4	^51^	Single wall CNT	2600	76.5	531.8	Cylindrical

i. Spherical shaped nanoparticles:
  $$\frac{{\mu_{nf1} }}{{\mu_{bf} }} = 1 + 2.5\phi + 6.2\phi^{2} \,\,\,,\,\,\,\,\,\kappa_{nf1} = \kappa_{bf} \left[ {\frac{{2\kappa_{bf} + \kappa_{sp1} + 2\phi (\kappa_{sp1} - \kappa_{bf} )}}{{2\kappa_{bf} + \kappa_{sp1} - \phi (\kappa_{sp1} - \kappa_{bf} )}}} \right]$$.
ii. Cylindrical shaped nanoparticles:
  $$\frac{{\mu_{nf2} }}{{\mu_{bf} }} = 1 + 13.5\phi + 904.4\phi^{2} \,\,\,,\,\,\,\,\,\kappa_{nf2} = \kappa_{bf} \left[ {\frac{{3.9\kappa_{bf} + \kappa_{sp2} + 3.9\phi (\kappa_{sp2} - \kappa_{bf} )}}{{3.9\kappa_{bf} + \kappa_{sp2} - \phi (\kappa_{sp2} - \kappa_{bf} )}}} \right]$$
iii. Platelet shaped nanoparticles:
  $$\frac{{\mu_{nf3} }}{{\mu_{bf} }} = 1 + 37.1\phi + 612.6\phi^{2} \,\,\,,\,\,\,\,\,\kappa_{nf3} = \kappa_{bf} \left[ {\frac{{4.7\kappa_{bf} + \kappa_{sp3} + 4.7\phi (\kappa_{sp3} - \kappa_{bf} )}}{{4.7\kappa_{bf} + \kappa_{sp3} - \phi (\kappa_{sp3} - \kappa_{bf} )}}} \right]$$

### Similarity transformations

The existence of the stream function $\psi (x,y)$ is considered as,

23 $$U = \frac{\partial \psi }{{\partial Y}}\;{\text{and}}\;V = - \frac{\partial \psi }{{\partial X}}.$$

And the similarity variables are

24 $$\psi = X\,f(Y)\,,\,\,N = X\,g(Y)\,\,,\,\,\,T = \Theta (Y)\,,\,\,C = \Phi (Y)$$

Using Eqs. (23) and (24) to solve Eqs. (16)–(20), we obtain the following ODEs

25 $$\left( {1 + \frac{{Er\,A_{3} }}{{A_{1} }}} \right)f^{\prime\prime}{^\prime}(Y)\, + f(Y)\,f^{\prime\prime}(Y) - f^{\prime 2} (Y) - \frac{{Da^{ - 1} A_{1} }}{{A_{2} }}f^{\prime}(Y) + \frac{{Er\,A_{3} }}{{A_{1} }}g^{\prime}(Y) = 0,$$

26 $$\left( {1 + \frac{{Er\,A_{3} }}{{2\,A_{1} }}} \right)g^{\prime\prime}(Y)\, + f(Y)g^{\prime}(Y) - f^{\prime}(Y)g(Y) - \frac{{Er\,A_{3} }}{{A_{1} }}\left[ {2g(Y) + f^{\prime\prime}(Y)} \right] = 0,$$

27 $$\left( {A_{3} + R} \right)\,\,\Theta ^{\prime\prime}(Y) + \frac{{A_{2} A_{4} \Pr }}{{A_{1} }}f(Y)\,\,\Theta ^{\prime}(Y) = 0,$$

28 $$\Phi ^{\prime\prime}(Y) + \frac{{A_{2} Sc}}{{A_{1} }}\left[ {f(Y)\,\Phi ^{\prime}(Y) - C_{r} \Phi (Y)} \right] = 0.$$

The corresponding boundary conditions are,

29 $$\left. {\begin{array}{llll} f(0) = V_{c}, & \quad f^{\prime}(0) = d, & \quad {} & \quad g(0) = - \xi f^{\prime\prime}(0) \\ \Theta (0) = 1\,\left( {{\text{for}}\,\,{\text{the}}\,{\text{PST}}} \right) & \quad {} & \quad {} & \quad \Theta ^{\prime}(0) = - 1\,\left( {{\text{for}}\,{\text{the}}\,{\text{PHF}}\,} \right) \\ \Phi (0) = 1\left( {{\text{for}}\,\,{\text{the}}\,{\text{PSC}}} \right), & \quad {} & \quad {} & \quad \Phi ^{\prime}(0) = - 1\,\left( {{\text{for}}\,{\text{the}}\,{\text{PMF}}\,} \right) \\ f^{\prime}(\infty ) = 0, & \quad g(\infty ) = 0, & \quad \Theta (\infty ) = 0 & \quad \Phi (\infty ) = 0. \\ \end{array} } \right\}$$

The non-dimensional parameters involved in Eq. (25)–(28) are:

$Er = \frac{{\kappa_{f} }}{{\mu_{f} }}$ is known as Eringen number,

$Da^{ - 1} = \frac{{\nu_{f} }}{a\,k}$ is known as inverse Darcy number.

The velocity component along the sheet is described as $u = U_{w} = dax$, such that $d > 0$ is the stretching parameter and $d < 0$ is the shrinking parameter and $d = 0$ represents permeability. The mass transpiration is defined as $V_{c} = - \frac{{{\text{v}}_{w} }}{{\sqrt {a\nu } }}$ in which $V_{c} > 0$ implies suction, $V_{c} < 0$ represents injection and $V_{c} = 0$ conveys no permeability.

The Prandtl number is denoted as $\Pr = \frac{{\nu_{f} }}{{\alpha_{f} }}$, radiation number is $R = \frac{{16\sigma *T_{\infty }^{3} }}{{3\kappa_{f} k*}}$, Schmidt number is denoted as $Sc = \frac{{\nu_{f} }}{D}$ and chemical reaction parameter is $C_{r} = \frac{{K_{1} }}{a}$.

Further,

$$A_{1} = \frac{{B_{1} \phi_{1} + B_{2} \phi_{2} + B_{3} \phi_{3} }}{\phi }$$, $$A_{2} = 1 - \phi_{1} - \phi_{2} - \phi_{3} + \phi_{1} \frac{{\rho_{sp1} }}{{\rho_{f} }} + \phi_{2} \frac{{\rho_{sp2} }}{{\rho_{f} }} + \phi_{3} \frac{{\rho_{sp3} }}{{\rho_{f} }}$$,

$$A_{3} = \frac{{B_{1} \phi_{1} + B_{2} \phi_{2} + B_{3} \phi_{3} }}{\phi }$$, $$A_{4} = 1 - \phi_{1} - \phi_{2} - \phi_{3} + \phi_{1} \frac{{\left( {\rho c_{p} } \right)_{sp1} }}{{\left( {\rho c_{p} } \right)_{f} }} + \phi_{2} \frac{{\left( {\rho c_{p} } \right)_{sp2} }}{{\left( {\rho c_{p} } \right)_{f} }} + \phi_{3} \frac{{\left( {\rho c_{p} } \right)_{sp3} }}{{\left( {\rho c_{p} } \right)_{f} }}$$,

$$B_{1} = 1 + 2.5\phi + 6.2\phi^{2}$$, $$B_{2} = 1 + 13.5\phi + 904.4\phi^{2}$$ , $$B_{3} = 1 + 37.1\phi + 612.6\phi^{2}$$, $$B_{4} = \left[ {\frac{{\kappa_{sp1} + 2\kappa_{f} - 2\phi (\kappa_{f} - \kappa_{sp1} )}}{{\kappa_{sp1} + 2\kappa_{f} + \phi (\kappa_{f} - \kappa_{sp1} )}}} \right]$$ , $$B_{5} = \left[ {\frac{{\kappa_{sp2} + 3.9\kappa_{f} - 3.9\phi (\kappa_{f} - \kappa_{sp2} )}}{{\kappa_{sp2} + 3.9\kappa_{f} + \phi (\kappa_{f} - \kappa_{sp2} )}}} \right]$$ and $$B_{6} = \left[ {\frac{{\kappa_{sp3} + 4.7\kappa_{f} - 4.7\phi (\kappa_{f} - \kappa_{sp3} )}}{{\kappa_{sp3} + 4.7\kappa_{f} + \phi (\kappa_{f} - \kappa_{sp3} )}}} \right]$$.

## Solution analysis

### Exact solution for momentum and microrotation

The analytical solutions for momentum Eq. (25) and microrotation Eq. (26) when subjected to boundary conditions Eq. (29) are as follows (Mahabaleshwar et al.^52^):

30 $$f(Y) = V_{c} + \frac{d}{\delta }\left[ {1 - Exp( - \delta \eta )} \right],$$

31 $$g(Y) = d\,\xi \,\delta \,Exp( - \delta Y).$$

Using Eqs. (30) and (31), we solve the Eqs. (25) and (26) to obtain the following algebraic equation:

32 $$\left[ {1 + \frac{{Er\,A_{3} }}{{A_{1} }}(1 - \xi )} \right]\delta^{2} - V_{c} \delta - \left( {d + \frac{{A_{1} \,Da^{ - 1} }}{{A_{2} }}} \right) = 0,$$

33 $$\left( {1 + \frac{{Er\,A_{3} }}{{2\,A_{1} }}} \right)\xi \,\delta^{2} - V_{c} \,\xi \,\delta \, + \left[ {\frac{{Er\,A_{3} }}{{A_{1} }}(1 - 2\xi ) - d\xi } \right] = 0.$$

Here, $\xi$ is the boundary constant which lies between 0 and 1. When $\xi = 0$, it implies a strong concentration of microelements at the sheet while $\xi = \frac{1}{2}$ represents a weak concentration, where as $\xi = 1$ is used to represent the turbulent flow of fluid.

The algebraic equation in Eq. (32) has the following zeros:

34 $$\left. \begin{gathered} \delta_{1} = \frac{{V_{c} + \sqrt {4pq + V_{c}^{2} } }}{2p} \hfill \\ \delta_{2} = \frac{{V_{c} - \sqrt {4pq + V_{c}^{2} } }}{2p} \hfill \\ \end{gathered} \right\}\,\,\,\,\,\,{\text{for}}\,\,\,\xi \,{ = }\frac{1}{2},$$

where,

$$p = \left[ {1 + \frac{{Er\,A_{3} }}{{A_{1} }}(1 - \xi )} \right]$$

and

$$q = \left[ {d + \frac{{A_{1} \,Da^{ - 1} }}{{A_{2} }}} \right].$$

Here, existence of the unique solution for $d = 1$(stretching sheet) and dual solutions exists for $d = - 1$(shrinking sheet) are determined. Also, in Eq. (34), $\delta_{1}$ corresponds to the UB solution and $\delta_{2}$ corresponds to the LB solution. Note that $Da^{ - 1} \,,\,\,Er\,\,{\text{and}}\,\,\delta$ parameters are negative in order to satisfy the boundary condition far from the wall.

### Quantities of physical interest

The non-dimensional coefficient of skin friction is defined as,

35 $$\sqrt {\text{Re}} \,C_{f\,x} = \,\left[ {\frac{{A_{1} + (1 - \xi )\,Er\,A_{3} }}{{A_{2} }}} \right]\,f^{\prime\prime}(0),$$

where,

36 $$C_{f\,x} = \frac{{\left[ {(\mu_{tnf} + \kappa_{tnf} )\left( {\frac{\partial u}{{\partial y}}} \right) + \frac{{\kappa_{tnf} }}{a}N} \right]_{y = 0} }}{{\rho_{tnf\,} u_{w}^{2} }},$$

and, ${\text{Re}}_{x} = \frac{{a\,x^{2} }}{{\nu_{f} }}$ represent local Reynolds number.

### Solution of the temperature equation

Rewriting Eq. (27) by substituting $f(Y) = V_{c} + \frac{d}{\delta }\left[ {1 - Exp( - \delta \eta )} \right]$,

we get,

37 $$\left( {A_{3} + R} \right)\,\,\Theta ^{\prime\prime}(Y) + \frac{{A_{2} A_{4} \Pr }}{{A_{1} }}\left( {V_{c} + \frac{d}{\delta }\left[ {1 - Exp( - \delta \eta )} \right]} \right)\,\,\Theta ^{\prime}(Y) = 0.$$

Now, introducing a new variable $\vartheta_{1} = \left( {\frac{d\,\Pr }{{\delta^{2} }}} \right)Exp[ - \delta Y]$ and substituting in Eq. (37),

we get,

38 $$\vartheta_{1} \frac{{d^{2} \Theta }}{{d\vartheta_{1} }} + \left[ {1 - b_{1} + c_{1} \,\vartheta_{1} } \right]\frac{d\Theta }{{d\vartheta_{1} }} = 0,$$

where,

$$b_{1} = \frac{{A_{4} \Pr \,V_{c} (\delta + d)}}{{\delta^{2} (A_{3} + R)}}$$

and

39 $$c_{1} = \frac{{A_{3} \Pr }}{{A_{3} + R}}.$$

With the corresponding imposed boundary conditions

40 $$\Theta (\vartheta_{1} = 0) = 0,\,\,\,\,\,\,\Theta \left( {\vartheta_{1} = \frac{d\,\Pr }{{\delta^{2} }}} \right) = 1\,({\text{PSH}}\,{\text{case}})\,\,\,\,{\text{and}}\,\,\,\,\Theta ^{\prime}\left( {\vartheta_{1} = \frac{d\,\Pr }{{\delta^{2} }}} \right) = - 1\,({\text{PHF}}\,{\text{case}}).$$

The analytical solution to the heat equation for both PSH and PHF cases is derived in terms of incomplete gamma function.

41 $$\Theta (\eta ) = \frac{{\Gamma [b_{1} ,0] - \Gamma \left( {b_{1} ,\,\,\frac{{c_{1} \,d\Pr }}{{\delta^{2} }}\,Exp[ - \delta Y]} \right)}}{{\Gamma [b_{1} ,0] - \Gamma \left( {b_{1} ,\,\,\frac{{c_{1} \,d\Pr }}{{\delta^{2} }}\,} \right)}}\;\left( {\text{PSH case}} \right),$$

42 $$\Theta (Y) = \frac{{\Gamma [b_{1} ,0] - \Gamma \left( {b_{1} ,\,\,\frac{{c_{1} \,d\Pr }}{{\delta^{2} }}\,Exp[ - \delta Y]} \right)}}{{\delta \,Exp\left[ {\frac{{ - c_{1} \,d\,\Pr }}{{\delta^{2} }}} \right]\left( {\frac{{c_{1} \,d\Pr }}{{\delta^{2} }}} \right)^{b} }}\;\;\left( {\text{PHF case}} \right).$$

### Solution of the concentration equation

Rewriting Eq. (28) by substituting $f(Y) = V_{c} + \frac{d}{\delta }\left[ {1 - Exp( - \delta \eta )} \right]$,

43 $$\Phi ^{\prime\prime}\,(Y) + \frac{{A_{2} Sc}}{{A_{1} }}\left( {V_{c} + \frac{d}{\delta }\left[ {1 - Exp( - \delta \eta )} \right]} \right)\,\Phi ^{\prime}\,(Y) = 0.$$

Now, introducing a new variable $\vartheta_{2} = \left( {\frac{d\,Sc}{{\delta^{2} }}} \right)Exp[ - \delta Y]$ and substituting in Eq. (43),

we get,

44 $$\vartheta_{2} \frac{{d^{2} \Theta }}{{d\vartheta_{2} }} + \left[ {1 - b_{2} + c_{2} \,\vartheta_{2} } \right]\frac{d\Theta }{{d\vartheta_{2} }} = 0,$$

where,

45 $$b_{2} = \frac{{A_{4} \Pr \,V_{c} (\delta + d)}}{{\delta^{2} (A_{3} + R)}}\,\,\,\,\,{\text{and}}\,\,\,\,c_{2} = \frac{{A_{3} Sc}}{{A_{3} + R}}.$$

With the corresponding boundary conditions:

46 $$\Theta (\vartheta_{2} = 0) = 0,\,\,\,\Theta \left( {\vartheta_{2} = \frac{d\,Sc}{{\delta^{2} }}} \right) = 1\,({\text{PSH}}\,{\text{case}})\,\,\,{\text{and}}\,\,\,\Theta ^{\prime}\left( {\vartheta_{2} = \frac{d\,Sc}{{\delta^{2} }}} \right) = - 1\,({\text{PHF}}\,{\text{case}})\,\,$$

The analytical solution to the heat equation for both PSH and PHF cases is derived in terms of incomplete gamma function.

47 $$\Phi (\eta ) = \frac{{\Gamma [b_{2} ,0] - \Gamma \left( {b_{2} ,\,\,\frac{{c_{2} \,d\,Sc}}{{\delta^{2} }}\,Exp[ - \delta Y]} \right)}}{{\Gamma [b_{2} ,0] - \Gamma \left( {b_{2} ,\,\,\frac{{c_{2} \,d\,Sc}}{{\delta^{2} }}\,} \right)}}\;\left( {\text{PSH case}} \right)$$

48 $$\Phi (Y) = \frac{{\Gamma [b_{2} ,0] - \Gamma \left( {b_{2} ,\,\,\frac{{c_{2} \,d\,Sc}}{{\delta^{2} }}\,Exp[ - \delta Y]} \right)}}{{\delta \,Exp\left[ {\frac{{ - c_{2} \,d\,Sc}}{{\delta^{2} }}} \right]\left( {\frac{{c_{2} \,d\,Sc}}{{\delta^{2} }}} \right)^{b} }}\;\left( {\text{PHF case}} \right)$$

## Result analysis

**Figure 2:**
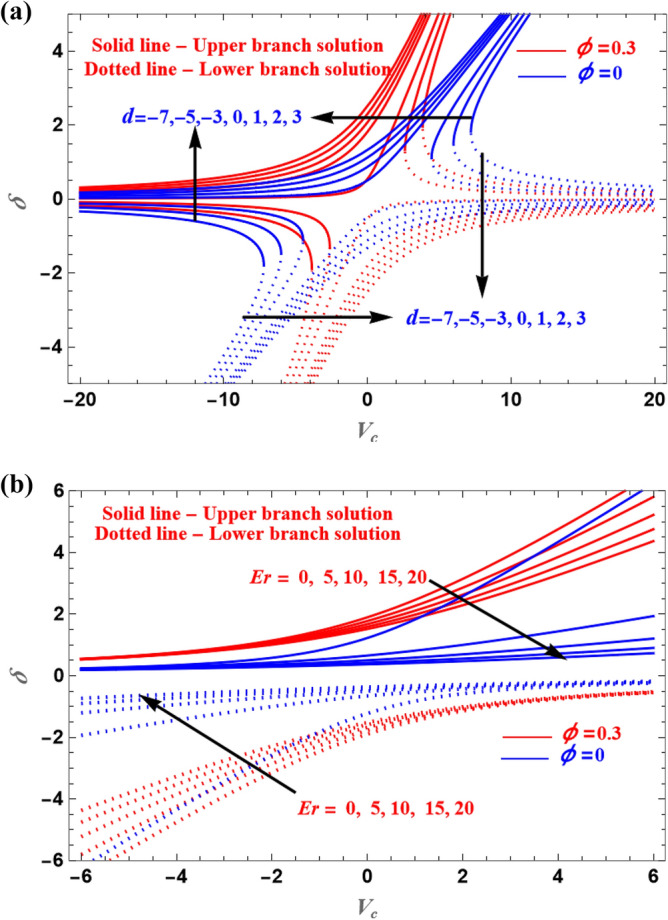
(**a**,**b**) Illustration of the solution for various values of $d$ and $Er$ parameters.

The boundary layer flow of the micropolar fluid, which is infused with ternary nanoparticles is analysed in this problem. The thermal conductivity and mass transfer are noted in this analysis. Solution plots are represented through graphs for various parameters. Furthermore, the current work's results are analysed for the presence of nanoparticles and compared to the absence of nanoparticles. Results of the presence and absence of the nanoparticles are shown in the graphs using red lines (for the presence of nanoparticles) and blue lines (for the absence of nanoparticles). The solution domain of $\delta$ is plotted versus $V_{c}$ for distinct values of $d$ and $Er$, respectively, is shown in Fig. 2a,b. While decreasing the LB solution, increasing value of $d$ increases the UB solution. The LB solution is increased while the UB solution is decreased as the parameters $Er$ and $V_{c}$ is increased. This is clear from Fig. 2 that is highly dependent on the variables $V_{c}$, $Er$, and $d$.

**Figure 3:**
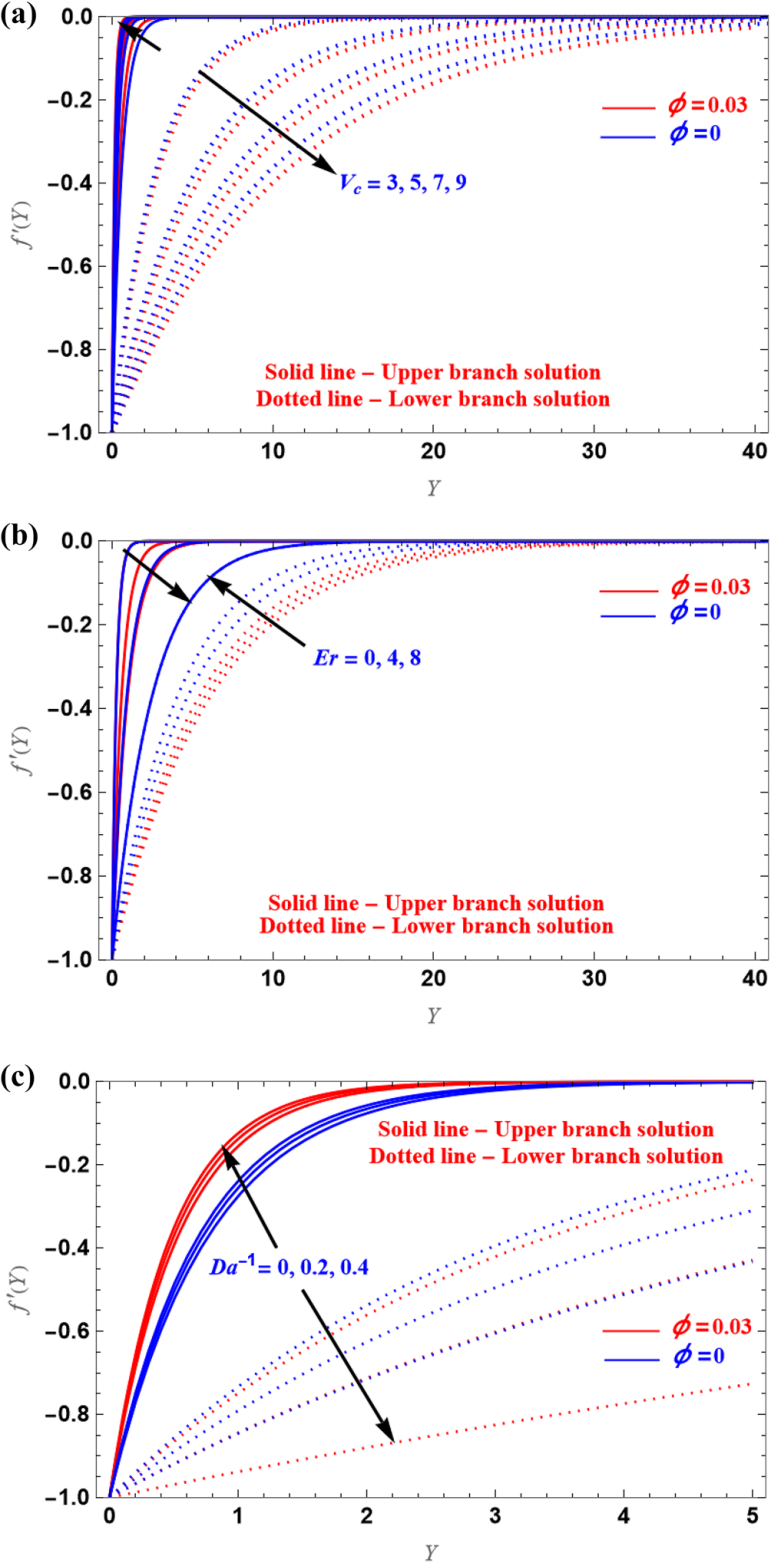
(**a**–**c**) Axial velocity plot for various physical parameters.

The impacts of the $V_{c}$(suction), $Er$ and $Da^{ - 1}$ are examined in the axial velocity profile, as shown in Fig. 3a–c. The UB velocity increased as $V_{c}$ and $Da^{ - 1}$ is increased while the LB velocity decreases. However, $Er$ show the opposite trend. As a result, in every instance, the UB and LB solutions displayed opposing characteristics.

**Figure 4:**
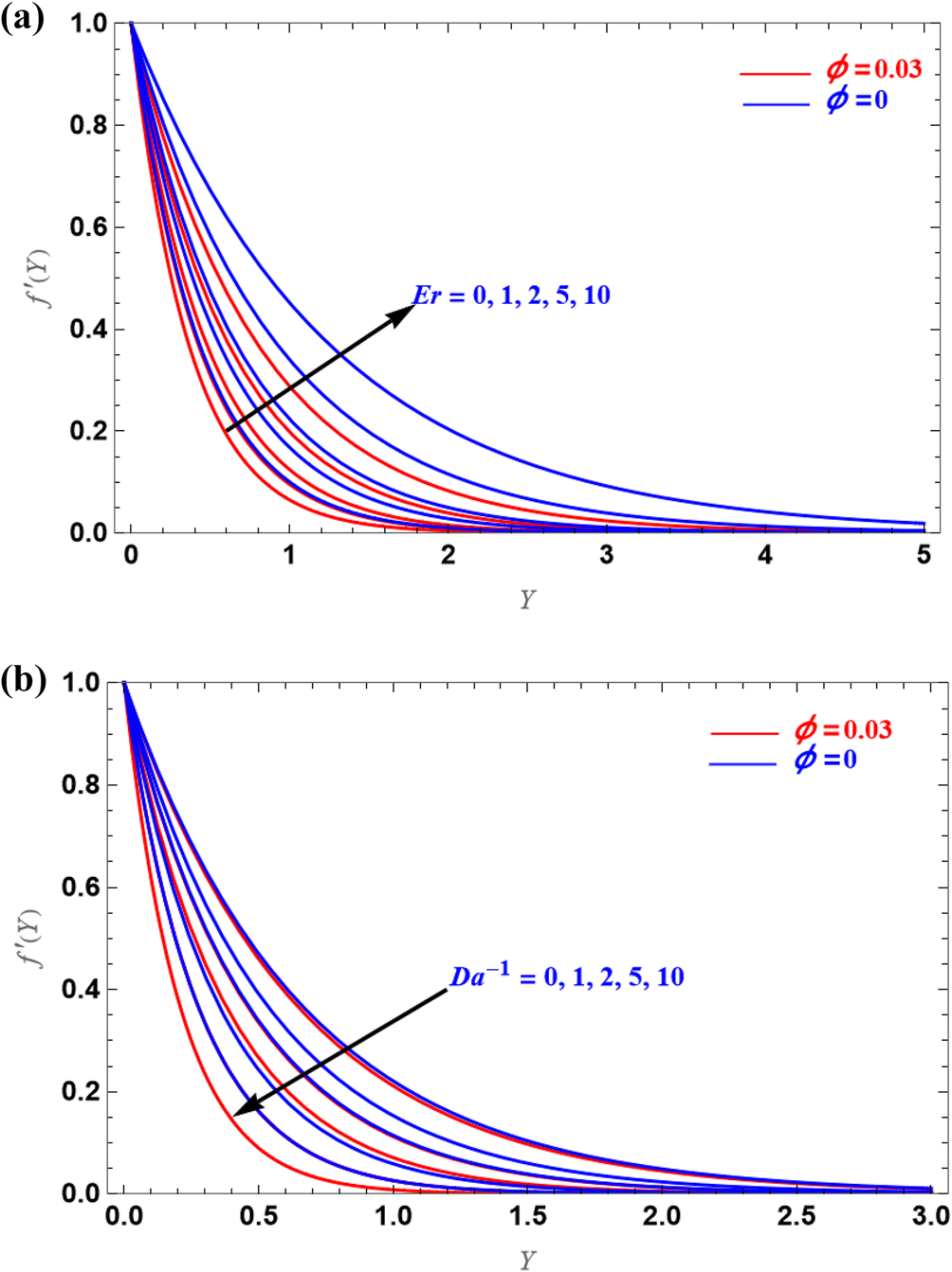
(**a**,**b**) Representation of $f^{\prime}(Y)$ versus $Y$.

In the case of the stretching, the axial velocity profiles are shown in Fig. 4a,b for several values of $Er$ and $Da^{ - 1}$. Since the viscous force and microrotation are produced due to non-Newtonian fluid, for large $Er$ value, velocity is enhanced, i.e., the boundary layer thickness is enhanced with the increased $Er$ values. As can be seen from Fig. 4b, the inverse behaviour was observed in the case of the $Da^{ - 1}$.

**Figure 5:**
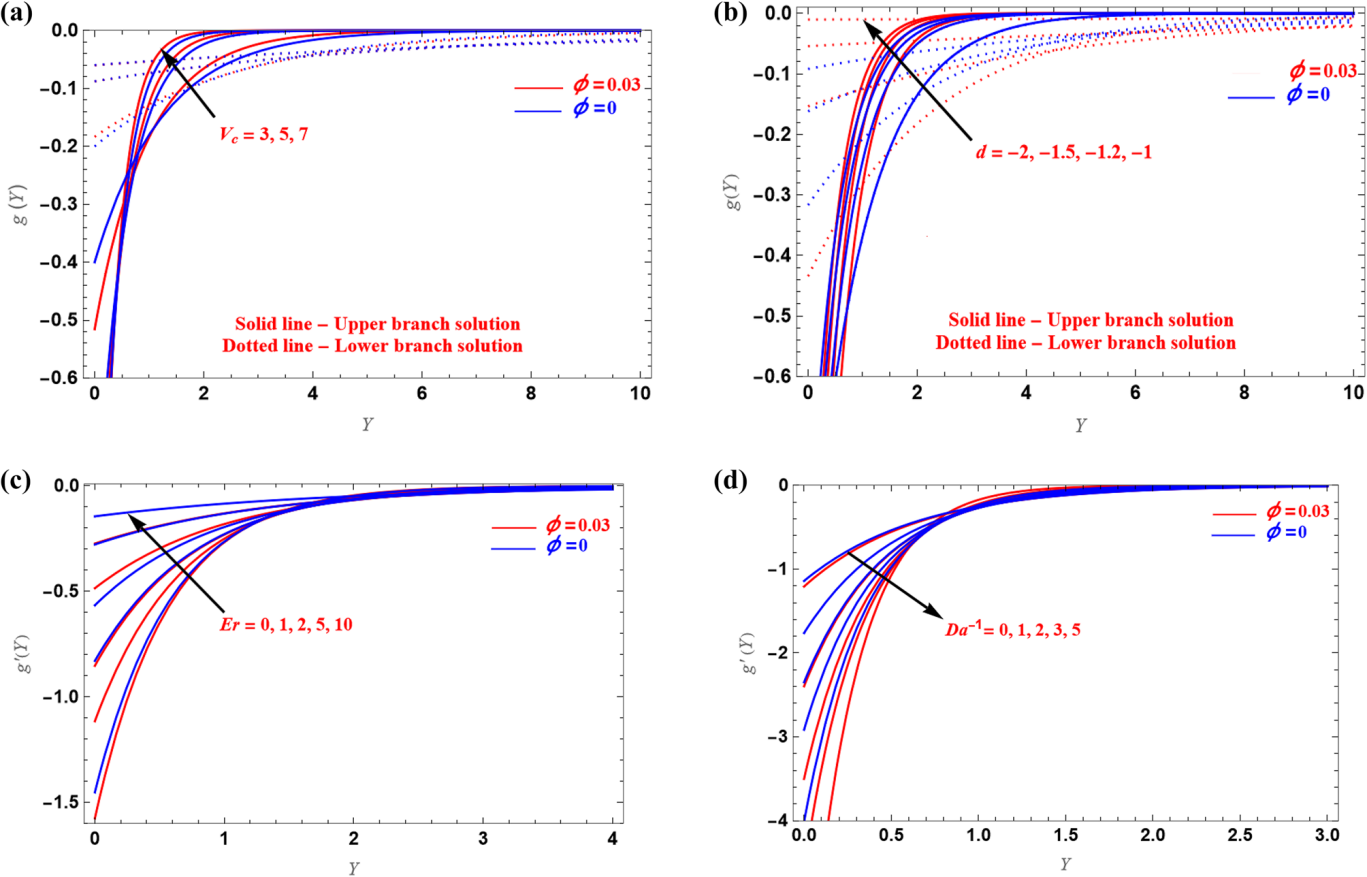
(**a**–**d**) Illustration of $g(Y)$ verses $Y$ and $g^{\prime}(Y)$ verses $Y$.

The impact of the $V_{c} ( > 0)$ and $d( < 0)$ on $g(Y)$, $g^{\prime}(Y)$ profiles relative to $Y$ are shown in Fig. 5a,b for both UB and LB solutions. The microrotation in the UB increases due to the increased values of $d$ and $V_{c}$, while the microrotation tends to decrease in the LB case, as seen in Fig. 5a,b. Graphs of the $g^{\prime}(Y)$ against the similarity variable for different $Er$ and $Da^{ - 1}$ values in the stretching case are shown in Fig. 5c,d. When $Da^{ - 1}$ increases,$g^{\prime}(Y)$ decrease and $g^{\prime}(Y)$ increases with increase of $Er$ value. As a result, $Er$ and $Da^{ - 1}$ behave in opposing ways to $g^{\prime}(Y)$.

**Figure 6:**
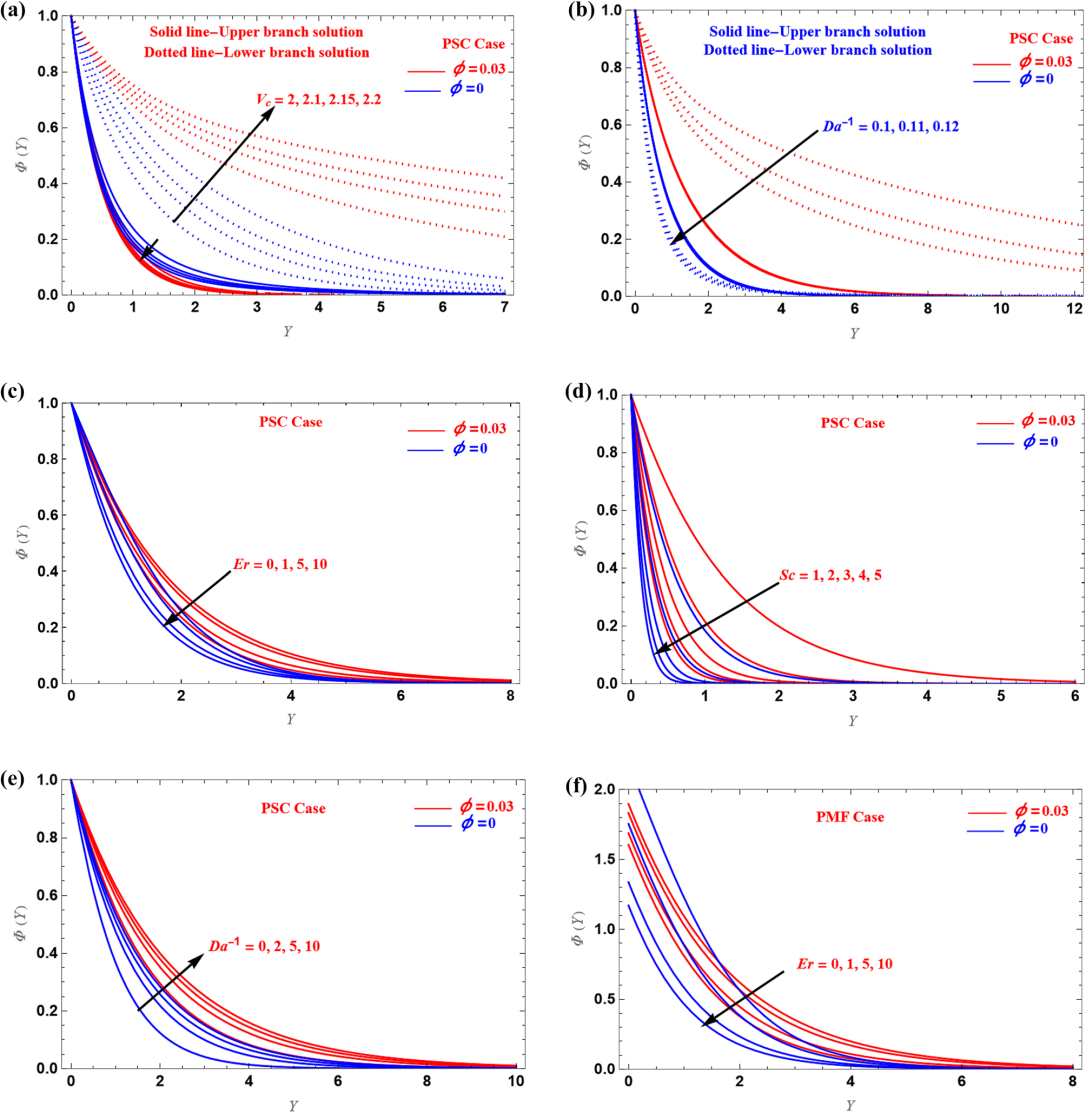

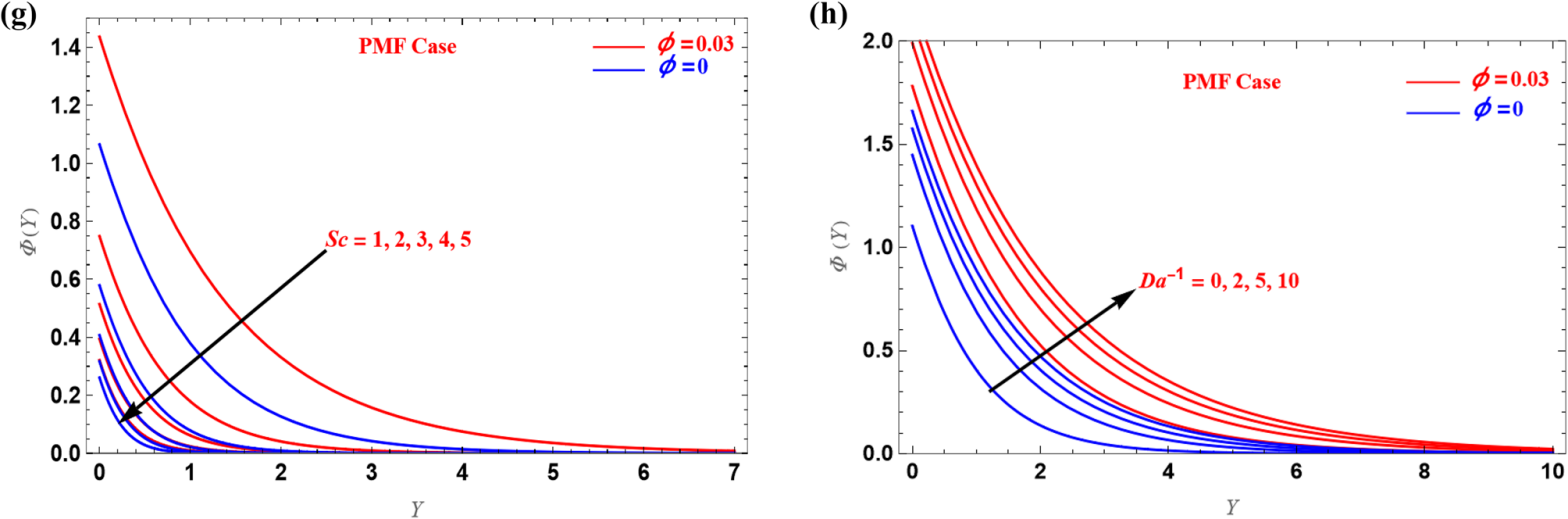
(**a**–**h**) Illustration of $\Phi (Y)$ versus $Y$.

For PSC and PMF cases, $\Phi (Y)$ profiles are shown in Fig. 6. Figure 6a,b show the shrinking sheet PSC cases for different values of $Da^{ - 1}$ and $V_{c}$. The concentration increases in the UB when we increased these parameters, but it opposite trend is observed in the LB. Figure c–e show the stretched sheet PSC examples, while Fig. 6e,f show the PMF cases. When the values $Er$, $Sc$ and $Da^{ - 1}$ are increased, the concentration increased in both instances. Therefore, these parameters have the effect of making the concentration boundary layer thicker. The boundary layer's temperature profile could be extremely important in solar heater applications.

**Figure 7:**
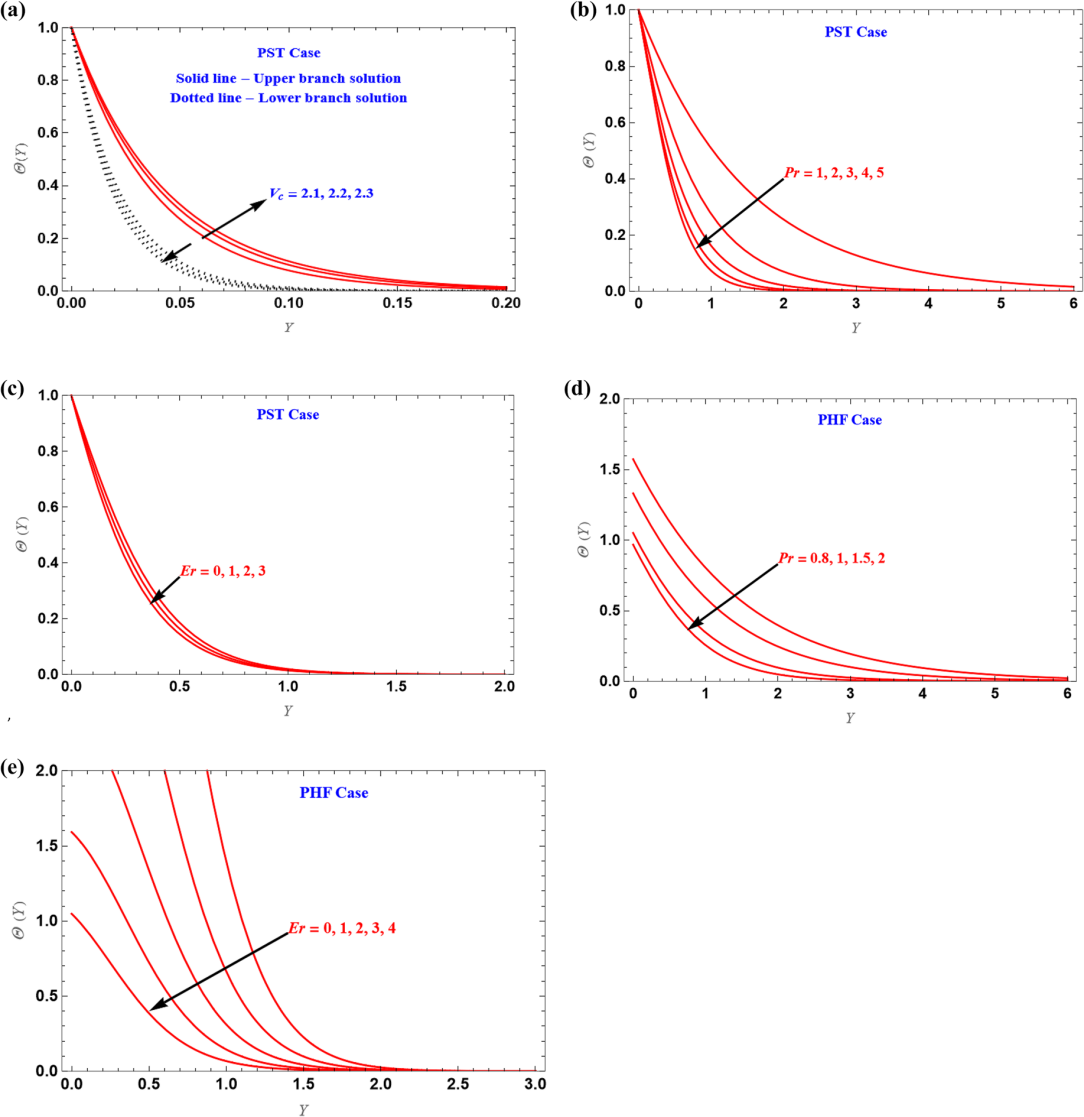
(**a**–**e**) Solution of heat versus similarity variable.

For different values of the $V_{c} ( > 0)$, *Pr* and *Er,* $\Theta (Y)$ is plotted for the PST and PHF cases, respectively, Fig. 7a–c illustrates the variation of temperature profiles. Due to the increased shear rate observed in this area, the effect of $V_{c} ( > 0)$ on the temperature profiles is significant near to the solid wall. Additionally, in the PSH situation, temperature profiles values decrease as $V_{c}$, *Pr* and *Er* values increase, similar effect is observed in the PHF Fig. 7d,e case also.

**Figure 8:**
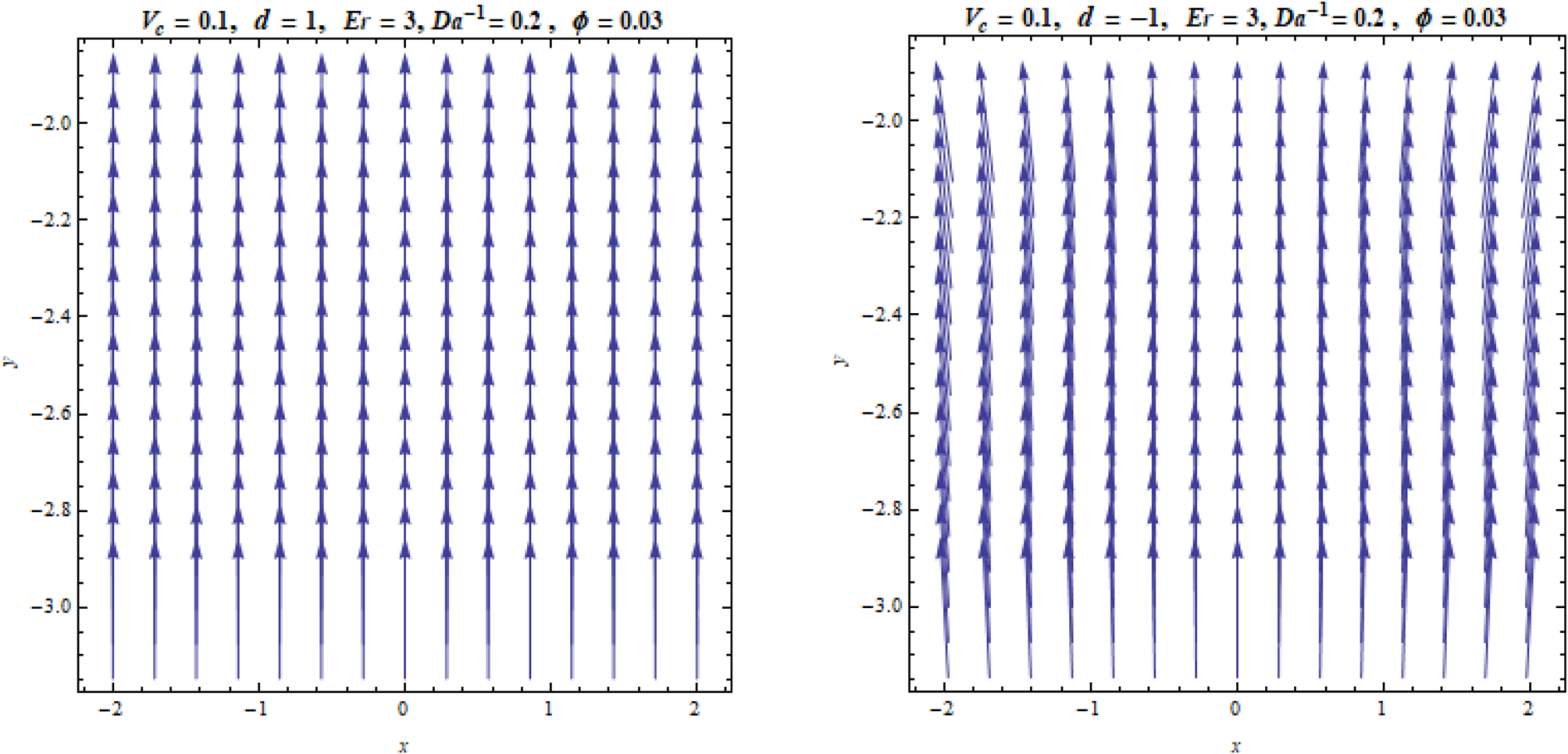
Streamlines representation of the flow.

Figure 8 shows the streamline flow pattern of boundary layer flow for the stretching and shrinking cases, respectively. It demonstrates that the nanoparticles’ paths are straight, and a tangent made to one of them at any point reveals the direction in which the liquid is moving at that location. In the case of shrinking, the fluid flow is moving adjacent to the surface faster when compared with their movement in the stretching case.

## Concluding remarks

The boundary layer flow of micropolar fluid that is infused with ternary nanoparticles is analysed in this study. Due to the inclusion of ternary nanoparticles, enhanced thermal conductivity is observed in this analysis. Solution plots are represented through graphs for various parameters. The following are the results noted in this study:

- The UB velocity increased as $V_{c}$ and $Da^{ - 1}$ is increased while the LB velocity decreases while, increased values of $Er$ is decreasing the UB velocity and increasing LB velocity.
- In the case of the stretching, for large $Er$ value, velocity is enhanced. But there seems to be velocity drop in the case of the $Da^{ - 1}$.
- The impact of the $V_{c} ( > 0)$ and $d( < 0)$ are greatly influencing $g(Y)$, $g^{\prime}(Y)$ profiles. The microrotation in the UB increases due to the increased values of $d$ and $V_{c}$, while the microrotation tends to decrease in the LB case.
- For shrinking sheet, concentration is increased for increased values of $Da^{ - 1}$ and $V_{c} ,$ while for stretching sheet, concentration is decreased for PSC case for *Sc* and $Er$ parameters but observed increasing for increasing values of $Da^{ - 1}$ for PMF case.
- The existence of dual solutions is found for velocity, microrotation, and concentration in the case of a shrinking sheet and the existence of unique solution is observed for the stretching sheet.

## Data Availability

The datasets used and/or analysed during the current study available from the corresponding author on reasonable request.

## Latin symbols

*a*: Constant (s^−1^)
$$c,C$$: Concentration parameters $$\left( {{\text{mol m}}^{ - 3} \,} \right)$$
$C_{r}$: Chemical reaction parameter $\left( { = \frac{{K_{1} }}{a}} \right)$ $\left( - \right)$
$$C_{w}$$: Wall concentration $$\left( {{\text{mol m}}^{ - 3} \,} \right)$$
$d$: Shrinking/stretching parameter $\left( - \right)$
$$D$$: Diffusivity coefficient $$\left( - \right)$$
$Da^{ - 1}$: Inverse Darcy number $\left( { = \frac{\nu }{a\,k}} \right)$ $\left( - \right)$
$$Er$$: Eringen constant $$\left( {{\text{m}}^{2} \;{\text{s}}^{ - 1} } \right)$$
$$f$$: Similarity variable $$\left( - \right)$$
$g$: Non-dimensional microrotation function $\left( - \right)$
$$J$$: Microinertia $$\left( { = \frac{{\nu_{tnf} }}{a}} \right)$$ $$({\text{m}}^{2} )$$
$$k$$: Permeability $$\left( {{\text{N}}\,{\text{A}}^{ - 2} } \right)$$
$k^{*}$: Average absorption coefficient $\left( - \right)$
$K_{1}$: Chemical reaction coefficient $\left( - \right)$
$m_{w}$: Fluid mass at the boundary $\left( {{\text{kg}}\,{\text{s}}^{ - 1} } \right)$
$$N$$: Angular velocity $$\left( {{\text{rad}}\,{\text{s}}^{ - 1} } \right)$$
*p*: Pressure $$\left( {{\text{Pascel}}} \right)$$
*Pr*: Prandtl number $\left( { = \frac{\nu }{\alpha }} \right)$ $\left( - \right)$
$q_{r}$: Radiant heat flow $\left( {{\text{W}}\;{\text{m}}^{ - 2} } \right)$
$q_{w}$: Wall heat flux $\left( {{\text{W}}\;{\text{m}}^{ - 2} } \right)$
$$R$$: Radiation parameter $$\left( { = \frac{{16\,\sigma *T_{\infty }^{3} }}{3\kappa k*}} \right)$$ $$\left( - \right)$$
$${\text{Re}}_{x}$$: Reynolds number $$\left( { = \frac{{a\,x^{2} }}{\nu }} \right)$$ $$\left( - \right)$$
$$Sc$$: Schmidt number $$\left( { = \frac{\nu }{D}} \right)$$ $$\left( - \right)$$
$$t,T$$: Temperature $$\left( {\text{K}} \right)$$
$$T_{w}$$: Wall temperature $$\left( {\text{K}} \right)$$
$T_{\infty }$: Temperature far from the wall $\left( {\text{K}} \right)$
$$u,v$$: Velocity components $$\left( {{\text{m}}^{2} {\text{s}}^{ - 1} } \right)$$
$U,V$: Non-dimensional velocity components $\left( - \right)$
$x,y$: Co-ordinates of the 2-D plane $\left( {\text{m}} \right)$
$X,Y$: Non-dimensional co-ordinates $\left( - \right)$

## Greek symbols

$$\alpha$$: Thermal diffusivity $$\left( { = \frac{\kappa }{{\rho \,C_{p} }}} \right)$$ $$\left( {{\text{m}}^{2} {\text{s}}^{ - 1} } \right)$$
$$\delta$$: Parameters $$\left( - \right)$$
$\gamma_{tnf}$: Angular rotational viscosity $\left( {{\text{m}}^{2} {\text{s}}^{ - 1} } \right)$
$$\varepsilon$$: Porosity parameter $$\left( - \right)$$
$$\kappa$$: Thermal conductivity $$\left( {{\text{W}}\;{\text{m}}^{ - 1} {\text{K}}^{ - 1} } \right)$$
$$\mu$$: Dynamic viscosity $$\left( {{\text{kg}}\;{\text{m}}^{ - 1} {\text{s}}^{ - 1} } \right)$$
$$\nu$$: Kinematic viscosity $$\left( { = \frac{{\mu_{bf} }}{{\rho_{bf} }}} \right)$$ $$\left( {{\text{m}}^{2} {\text{s}}^{ - 1} } \right)$$
$$\rho$$: Density (kg m^−3^)
$\sigma *$: Stefan-Boltzmann constant (W m^-2^ K^-4^)
$$\Theta$$: Temperature function $$\left( { = \frac{{T - T_{\infty } }}{{T_{w} - T_{\infty } }}} \right)$$ $$\left( - \right)$$
$\phi$: Volume fraction of particle $\left( {0 < \phi < 1} \right)$ $\left( - \right)$
$$\xi$$: Parameter $$\left( - \right)$$
$$\Phi$$: Concentration function $$\left( { = \frac{{C - C_{\infty } }}{{C_{w} - C_{\infty } }}} \right)$$ $$\left( - \right)$$
$$\Psi$$: Stream function $$\left( - \right)$$

## Subscripts

$w$: At the boundary $\left( - \right)$
$\infty$: Far from the sheet $\left( - \right)$
$f$: Notation for base fluid $\left( - \right)$
$tnf$: Ternary nanofluid notation $\left( - \right)$

## Abbreviations

2-D: Two-dimensional $$\left( - \right)$$
ODEs: Ordinary differential equations $\left( - \right)$
PDEs: Partial differential equations $\left( - \right)$
PMF: Prescribed mass flux $\left( - \right)$
PSC: Prescribed surface concentration $\left( - \right)$
PST: Prescribed surface temperature $\left( - \right)$
PHF: Prescribed heat flux $\left( - \right)$
THNF: Ternary hybrid nanofluid $\left( - \right)$
MHD: Magnetohydrodynamics $$\left( - \right)$$
UB: Upper branch $\left( - \right)$
LB: Lower branch $\left( - \right)$
ADM: Adomian decomposition method $\left( - \right)$
